# Unlocking the power of implementation research

**DOI:** 10.1186/s44263-025-00171-9

**Published:** 2025-12-10

**Authors:** Michael J. Penkunas, Phyllis Dako-Gyeke, Nancy Gore Saravia, Yodi Mahendradhata, Adama Faye, Sanjay Rampal, Bakhyt Sarymsakova, Wafa Kammoun Rebai, John Reeder, Mahnaz Vahedi, Anna Thorson

**Affiliations:** 1 Special Programme for Research and Training in Tropical Diseases (TDR), WHO, Geneva, Switzerland; 2 Department of Social and Behavioural Sciences, School of Public Health, College of Health Sciences, University of Ghana, Accra, Ghana; 3 Centro Internacional de Entrenamiento E Investigaciones Médicas, CIDEIM, Cali, Colombia; 4 Universidad Icesi, Cali, Colombia; 5 Department of Health Policy and Management, Faculty of Medicine, Public Health and Nursing, Universitas Gadjah Mada, Yogyakarta, Indonesia; 6 Institute of Health and Development (ISED), Cheikh Anta Diop University, Dakar, Senegal; 7 Centre of Epidemiology and Evidence Based Practice, Department of Social and Preventive Medicine, Faculty of Medicine, University Malaya, Kuala Lumpur, Malaysia; 8 Astana Medical University, Astana, Kazakhstan; 9 Regional Training Centre Supported by the Special Programme for Research and Training in Tropical Diseases (TDR), WHO, Pasteur Institute of Tunis, University of Tunis El Manar, Tunis, Tunisia

Implementation research (IR) holds exciting potential for improving the design and operation of health systems. Multi-sectoral participation and equitable partnerships grounded in problem solving for health impact promote the translation of the results of IR to practice and policy. Equitable access to IR training requires diverse and adaptive instruction.

## Increasing need for implementation research to solve global health challenges

The global health landscape is at a pivotal moment, where implementation research (IR)—the systematic approach to improving access to efficacious health interventions through understanding and alleviating implementation barriers—is increasingly recognized as a key to addressing challenges and bottlenecks in health systems [1]. IR continues to gain prominence as an essential tool for addressing gaps and inequities in healthcare delivery, as demonstrated through the growth of projects internationally and the emergence of specialized IR journals [2]. The maturation of IR has also driven intense interest in related training programmes and materials. In 2024 alone, 7300 people registered for the massive open online course on IR [3] and nearly 60,000 people accessed the online IR Toolkit [4] in 2023, both developed by the United Nations Children’s Fund/United Nations Development Programme/World Bank/World Health Organization Special Programme for Research and Training in Tropical Diseases (TDR). TDR’s training portfolio has evolved over the past 50 years and now includes, but is not limited to, freely available online courses, a library of toolkits and guides, short courses, mentored research funding, embedded research training programmes, as well as sponsorship for master's and doctoral level degrees.

In conjunction with *BMC Global and Public Health’s* commitment to prioritizing health programme implementation that promotes equity and practical utility (https://www.biomedcentral.com/collections/ISC), we advocate for increased emphasis on accessible, context-driven IR training in low- and middle-income countries (LMICs). We echo the journal’s call for community-led and contextually relevant research, elevating attention to the experiences of those directly affected by health system inefficiencies and implementation bottlenecks. Equitable, effective, and easily accessible IR training is imperative, particularly in the face of global health challenges, both historic and emerging [5]. Yet, learners in LMICs and health professionals within these health systems, who are arguably best positioned to identify and address implementation bottlenecks, often lack opportunities and access to supportive networks and IR education.

## Building IR capacity in LMICs through accessible training

To strengthen IR capacities of diverse stakeholders, different training modalities complementing university-based degree programmes are strategic for catering to a variety of entry points and professional roles through multiple forms and formats. Dedicated resources for applying IR as a problem-solving tool constitute a strategic investment in securing an IR workforce to help ensure health impact is achieved. Long-term financial engagements with training goals and milestones, as opposed to punctuated or project-based contracts, are needed to create enduring equitable partnerships. These efforts must not only expand access to training and funding opportunities but also support the decolonization of knowledge production through shifting power and authority in creating training tools towards local actors and organizations in LMICs. By prioritizing regionally driven research, stakeholder-led learning agendas, and co-constructed curricula, IR training itself becomes a tool for equity while addressing historical imbalances in health research.

Despite its growing popularity, the human capital needed to conduct high-quality IR in LMICs remains inadequate and this situation is compounded by the relative lack of top-down demand for IR from various stakeholders [6]. We believe that concerted efforts and equitable partnerships are vital to elevating the global profile of IR and its potential for health system improvement. The inclusion of governmental ministries, policymakers, and programme implementation entities in capacity strengthening efforts can maximize the potential impact of IR and its translation into actionable results. Likewise, knowledge transfer, including dissemination of evidence of the effectiveness and benefits of IR, together with the consolidation and triangulation of results, should be supported and institutionalized.

Multifaceted approaches are needed to increase access to IR training, while also enabling the process of forging IR leadership, not only in academic institutions but also within health systems [7]. Building equitable partnerships requires mutual effort, and is vital for global health programmes to succeed. Partnerships catalyze stakeholder engagement in context-specific IR projects, in forming networks of IR expertise, and in translating new knowledge into improved health service delivery. Funding for IR training and projects should ideally be an integral component of programme support, outside of specific funding calls [8].

## Keys to reframe training and support locally led IR

The next generation of IR will involve global, regional, and national networks, to ensure institutional development and support health, research, and educational leaders in building hands-on expertise, including standardization of core IR curricula and accessible tools for skill building within the health system [9]. To achieve this, concerted, dedicated, and sustained funding of IR and capacity building aiming for health impact in resource-limited settings is essential [10]. Enduring IR capacity is deeply rooted in co-created efforts by local stakeholders.

TDR has supported the development of a global network of Regional Training Centres to help address the pressing need for IR capacity in LMICs. These centres serve as regional hubs for advancing locally relevant IR training, fostering regional collaboration, and strengthening institutional capacity through hands-on learning opportunities and the distribution of research funding. The TDR Regional Training Centres aim to support regional and sub-regional networks and to seed a workforce of professionals within the health system who can evaluate existing evidence, pose meaningful IR questions, conduct high-quality IR, and translate findings into health policy and programme decisions.

Drawing on lessons learned from global training initiatives, experiences from the TDR-supported Regional Training Centres, and persistent challenges routinely identified by learners and health programme implementers, we present the following ten keys. These are not stand-alone suggestions; instead, they are a synthesis of the principles and needs discussed above. These keys are intended to reframe IR training through an equity lens and support locally led, participatory implementation research:

1. Collaborative design: engaging governments and a range of stakeholders, including the TDR-supported Regional Training Centres, ensures that IR curricula are culturally and practically relevant as well as responsive to national research priorities.
2. Contextual relevance: IR training tailored to global as well as regionally specific health challenges, diseases, and shortfalls will attract the attention of leadership and support the development of demand-driven IR projects.
3. Practical application: wherever possible, real-world application of IR concepts should be the focus of training rather than the acquisition of theoretical knowledge. Newly acquired skills can then be readily applied within health systems to ask and answer pressing IR questions.
4. Emphasis on team-based, interdisciplinary, and multi-sectoral research: learners from a wide range of disciplines beyond those with a direct connection to health, such as education, engineering, veterinary medicine, architecture, and agriculture, could be proactively incorporated into IR teams to acknowledge and address the multifaceted nature of global health challenges from a One Health perspective.
5. Competency through collective education: health practitioners, researchers, policymakers, community members, and social innovators should all be involved in designing training programmes; stakeholders beyond research specialists are needed to identify bottlenecks, disentangle contextual challenges, and develop solutions and tailored implementation strategies.
6. Accessible platforms and accreditation: online and hybrid learning techniques, where live or in-person sessions are combined with pre-recorded lectures or virtual classrooms, should be prioritized and made available in a range of languages to overcome geographical and resource challenges, and to support equitable access to training opportunities. Standardization of certificates, curricula, and methods for evaluating essential skills among learners enhances quality and recognition.
7. Sustainable and equitable partnerships: long-term partnerships are essential for experience-sharing, developing IR training content, stimulating networks, and spurring post-training research.
8. Continuous adaptation: training organizations are obliged to update their programmes regularly to maintain the state-of-the-art in IR methodologies and to adapt their training as global health priorities evolve.
9. Resource allocation: adequate levels of expertise and funding are essential to ensure the success of any IR training initiative. Hence, IR should be a routine line item for the application of these skills to contextualized implementation challenges of health programmes.
10. Monitoring and evaluation: IR training programmes should be routinely and rigorously evaluated to assess the impact of these initiatives, determine whether they are meeting their goals, and to inform the continuous adaptation process. These measures and outcomes should be developed and assessed collectively with stakeholders carrying out and learning from such programmes.

## Conclusions

Unlocking the potential of IR in global health requires a multifaceted, inclusive, and equity-focused approach. Training agendas driven by stakeholders ensure courses are responsive to regional needs and emphasize practical application. True transformation depends on shifts in power through equitable partnerships, sustained investment, and participatory approaches to learning. By joining forces, government actors, health professionals, academic institutions, and communities can prioritize locally led and owned IR capacity strengthening to create a pathway to decolonizing global health practices.

## Data Availability

No datasets were generated or analysed during the current study.
